# 日本におけるオンライン・ハラスメントの現状と対策：Twitterでの女性記者のツイート「炎上」を例に

**DOI:** 10.12688/f1000research.74657.2

**Published:** 2022-02-03

**Authors:** 礪波亜希 礪波, 吉田光男 吉田, 佐野幸恵 佐野

**Affiliations:** 1 Faculty of Business Sciences, University of Tsukuba, 3-29-1 Otsuka, Bunkyo, Tokyo, 112-0012, Japan; 2 Faculty of Engineering, Information and Systems, University of Tsukuba, 1-1-1 Tennodai, Tsukuba, Ibaraki, 305-8577, Japan

**Keywords:** ソーシャルメディア, オンライン・ハラスメント, Twitter, 政策, social media, online harassment, policy, COVID-19

## Abstract

ソーシャルメディア上の「炎上」はもはや社会問題といっても過言ではないが、日本語環境の炎上については、被害にあった当事者視点に基づいて検討した研究は稀少である。本稿では、オンライン・ハラスメントという概念を導入し、その現状を国内外の先行研究のレビューに基づいて概要を明らかにした。次に、Twitterで発生した女性記者ツイートの「炎上」事例を分析、ハラスメントを行うユーザーにはインフルエンサー群、インフルエンサーの犬笛に呼応する炎上加担ユーザー群、荒らしを行うユーザー群の三層があり、それぞれが異なる形で一人のユーザーに対してハラスメントを行っていたことを観察した。最後に、個人と組織が取るべき対策を述べる。

## Abstract (en)

Harassment on the Internet, particularly on social media such as Twitter, has reached a level where it can, without exaggeration, be characterised as a real-world societal problem in Japan. However, studies on this phenomenon in the Japanese language environment, especially adopting a victim-centric perspective, are rare. In this paper, we incorporated the concept of online harassment and reviewed existing studies about online harassment from Japan and abroad. We then conducted a detailed case analysis of the “flaming” of a female journalist and those who targeted her on Twitter. Based on our analysis, we observed that there were three layers of users who targeted the journalist: influencers, users who responded to the instigation by influencers, and trolls. Each harassed the journalist, but in a different manner. Given Japan’s particular difficulty of imposing domestic regulations on social media companies that are mostly from abroad, we propose and describe possible measures that individuals and their employers should consider taking.

## はじめに

近年、デジタル技術が進展し、ソーシャルメディア ^1^ (SNS) が普及することにより、多くの人が簡単に情報発信や収集を行うことが可能となった。また 2019 年に発生した新型コロナ感染症 (COVID-19) の感染拡大と、政府の緊急事態宣言を受け、できるだけ外出や対面を控えるため、人々がインターネットを利用する頻度も顕著に高まった （ 森下, 2021）。同時に、インターネット上のトラブル、とりわけSNS上におけるコミュニケーション不全に関する事件が多発している。いわゆる「ネット炎上」はもはや社会問題といっても過言ではない。「炎上」とは、ある人物が発言した内容や行った行為について、ソーシャルメディアに批判的なコメントが殺到する現象を意味する （ 山口, 2015）。

しかしながら、炎上に関する既存研究は、SNS 上における情報の現象について扱うものが多く、とりわけ日本語環境の SNS については、炎上の被害にあった当事者視点に基づいて検討した研究は稀少である。そこで本稿では、炎上現象を検討する際に、オンライン・ハラスメントという概念を導入し、「日本語環境におけるオンライン・ハラスメントの現状はどうなっているのか」という記述的問いを設定する。オンライン・ハラスメントとは、「インターネット上で行われる、扇情的なコメントやヘイトスピーチの繰り返しの投稿（「荒らし」「トロール」）、サイバーストーカー、身体的な脅迫、同意なしに性的に露骨な画像を公開すること（「リベンジポルノ」）、個人情報を公開する（「晒し」「ドクシング」）などの行為」を意味する ( PEN America, n.d.-b)。

以下ではまず、オンライン・ハラスメントの現状を先行研究のレビューに基づいて概要を明らかにし、次に、日本で約 5,500 万人が使用する SNS である Twitter で発生した、女性記者ツイートの「炎上」、およびオンライン・ハラスメントの事例分析を行う ( Statista Research Department, 2021)。最後に、個人と組織が取るべきオンライン・ハラスメント対策について述べる。なお本稿は質的研究であり、実証や仮説検証を目的とするものではない。

## オンライン・ハラスメントの現状

本稿の執筆時点において、オンライン・ハラスメントに関する既存研究は稀少である。学術データベース Web of Scienceで、すべてのテキストフィールドを対象に「オンライン・ハラスメント (online harassment)」を検索語として検索したところ、1,360 件の論文が該当した。2006 年に発表された、若者の「インターネット・ハラスメント」に関する小児科学の論文が、最初にオンライン・ハラスメントについて扱った学術論文のうちのひとつと考えられる ( Ybarra *et al.*, 2006)。他方、この数は、例えば「Twitter」という語で検索した結果30,225件に比すと格段に少ない。

オンライン・ハラスメントは、デジタル技術や SNS が社会にもたらした負の影響であるといえるが、既存のデジタル技術に関する研究はこうした負の変化に楽観的であったことも関係してか、この行為に特化した大規模な調査ですら多くはない ( Fung *et al.*, 2013; Jankowicz *et al.*, 2021)。また、オンライン・ハラスメントという問題のある現象が、後述するとおり2016年の米国大統領選挙が端緒となり、主に英語圏を中心に急速に認識されはじめたことから、調査分析の論考も政策志向であり、公的機関やNGOとの共著などで行われた政策提言を含む文書である傾向がある。なお、こうした研究はディスインフォメーション ^2^、オンライン虐待 (online abuse)、人権に関する研究領域で行われることが多い ( Jankowicz *et al*., 2021)。例えば、人権の観点からは、意見及び表現の自由に対する権利の促進と保護に関する国連特別報告者によって2021年7月に発表された「ジェンダー正義と意見・表現の自由の推進に関する報告書」でインターネット環境において特に女性に対して行われるハラスメントが取り上げられている ( Khan, 2021)。本邦においては、当初オンライン・ハラスメントは子どもの間のいじめがインターネット環境に移ったものと見なされていたようである（ 尚 *et al.*, 2018）。 申・濱田 (2021) では、女性地方議員 3 名に対する Twitter のリプライの分析が行われ、議員の発言や政治活動を否定、揶揄したり、議員の指示や応援を（執拗に）表明して議員の関心を引こうとするリプライの 2 種があったことが指摘された。

それでは、オンオンライン空間において、どのようなハラスメントがどの程度行われているのであろうか。以下では数少ない調査研究の結果をまとめることを試みる。

子どもの権利を推進する国際 NGO であるプラン・インターナショナルの 2020 年の調査によれば、日本の 15〜24 歳の若年女性 501 名のうち、25 %が「SNS で何らかの形でオンライン・ハラスメントを経験したことがある」と回答したという （ プラン・インターナショナル, 2020）。他方、31 カ国で行われた調査結果を比較したところ、各国でオンライン・ハラスメントの概念について理解が異なること、回答者によっては全く理解されていないことも明らかとなった。

米国・ピュー研究所による 2021 年の調査報告書によれば、米国人の 4 人に1人がオンライン・ハラスメントを受けたことがあり、このうち半数が政治的な意見を理由にハラスメントを受けたと答えた ( Vogels, 2021)。また、2014 年、2017 年の同様の調査に比して、身体的な脅迫や、ストーキング、継続的なハラスメント、セクシュアル・ハラスメントなど、より深刻なオンライン・ハラスメントを受けたと答えた人数が増加したという。

日本語環境については、インターネットプロバイダーの BIGLOBE 社による全国の 20 代 〜60 代の SNS を利用する男女 770 人に対する調査において、SNS で他者から誹謗中傷 ^3^をされたことがあるかとの質問に対し、4.5%が「よくある」、13%が「たまにある」と返答した （ BIGLOBE株式会社, 2020）。なかでも 20 代は年代別で最も顕著に誹謗中傷を受けた比率が高く、10%が「よくある」、18.9%が「たまにある」と回答したという。ただしここでの誹謗中傷はオンライン・ハラスメントの定義とは異なるため、注意が必要である。誹謗中傷とは、誹謗 （他人の悪口を言うこと） と中傷 （根拠のない悪口を言い、他人の名誉を傷つけること）の 2 つが合わさった語である （ 三省堂, 2013）。炎上とは、前述のとおり、誹謗中傷を含む批判的なコメントがソーシャルメディアに殺到する現象のことで、オンライン・ハラスメントは、誹謗中傷を含むハラスメント行為がオンライン上で行われることであり、時にその行為は集中的に行われ、炎上現象にもなり得る。 ^4^

オンライン・ハラスメントの全容を明らかにする試みとは別に、被害者の回復支援を優先したアプローチで行われた調査結果が存在する。文筆従事者の団体、国際ペンクラブの米国支部 PEN アメリカでは、記者やライターなどが SNS で深刻なハラスメントに遭っていることを重く見て、「オンライン・ハラスメント フィールドマニュアル」という調査研究事業を開始した ( PEN America, n.d.-c)。同団体が 2017 年に実施した調査によれば、オンライン・ハラスメントを受けた 230 名から回答があり（うち 196 名は性別の明記があり、136 名が女性、52 名が男性、8 名がその他）、インターネット上で発生したオンライン・ハラスメントが、被害者の実生活に深刻な影響を及ぼしていることが明らかとなった。例えば、67%の回答者が「自分や身近な人の身の危険を感じたり、作品の発表を控えたり、SNS のアカウントを永久に削除するなど、深刻な反応を示した」、64.3%が「ハラスメントを理由に SNS を休止した」、62.3%が「オンライン・ハラスメントが私生活や身体的、心理的、精神的な健康に影響を及ぼした」と答えている ( PEN America, n.d.-b)。また、ハラスメントを受けた理由としては、53.5%が「自分の政治的見解を述べたため」、「個人的な意見を述べたため」、38.9%が「性別や性自認を理由に標的にされた」、31%が「外見 (ルッキズム)」と回答しており、オンライン・ハラスメントが SNS 上で個人が意見表明を行ったこと、もしくは発言者のジェンダーやアイデンティティを理由に行われていることが分かる。

また、研究成果公開活動や、研究者同士のネットワーク拡大、また学生とのコミュニケーション促進の一助として、ソーシャルメディアの使用が奨励されている場合が多い大学界においても、研究者がオンライン・ハラスメントの被害に頻繁に遭い、有害な影響が出ていることが指摘されている。ゴッセら ( 2021)は、オンライン・ハラスメントは仕事、アイデンティティ、もしくは学者としての必要条件のように見なされているものの、ジェンダー、外見 （ルッキズム）の要素が絡まることで複雑化していると述べた。

ソビエラージ ( 2020) によれば、オンライン・ハラスメントは誰でも被害に遭う可能性があるが、特に加害者の標的になりやすい属性があるという。すなわち、

1) マイノリティである、
2) フェミニスト （旧来のジェンダー価値観に縛られない）である、
3) 政治、スポーツ、外交、防衛、サイバーセキュリティなど男性優位の領域について意見する

場合、ハラスメントの標的になりやすく、これらの属性の共通部分においては最もハラスメントに遭う確率が高くなる（ 図 1）。なお、欧州の調査では、女性議員の 58.2%が SNS 上でオンライン・ハラスメントを受けたことがあると返答したが、特に年齢の低さ（40歳以下）、次に野党党員であることなど、議会政治において少数派である側面がハラスメントに遭うリスクを高めるとの結果であった （ Inter-Parliamentary Union, 2018; 申・濱田, 2021）。

**図 1.**
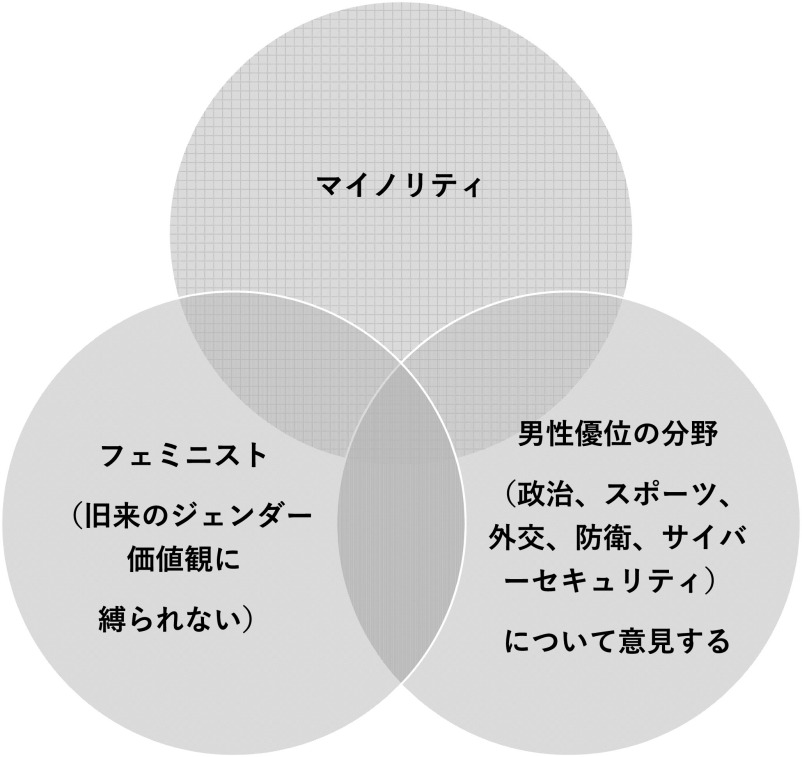
オンライン・ハラスメントの被害に遭いやすい属性 ( Jankowicz *et al* (2020)より).

米国のオンライン・ハラスメント被害者の支援団体であるオンライン SOS による報告書によると、オンライン上のハラスメントはインターネットの商業化が進んだ 1990 年代から存在していたが、2014~2016 年の「ゲーマーゲート」 ^5^ 期を経て、2016 年米国大統領選挙において一連のハラスメントの流れが確立した( Lee, 2019)。すなわち、悪意をもつ行為者が、様々な方策を使用し、多数の媒体を通じて、複数の場所で、標的 （被害者）に危害を与えるメカニズムが存在するのだという（ 図 2）。このように、最近の研究では、オンライン空間におけるハラスメント行為であっても被害者の受ける影響は甚大であること、またハラスメントや様々な暴力行為がオンライン空間ではなく、オフライン、すなわち現実世界に繋がることが問題視されている ( Berry, 2019)。 ^6^

**図 2.**
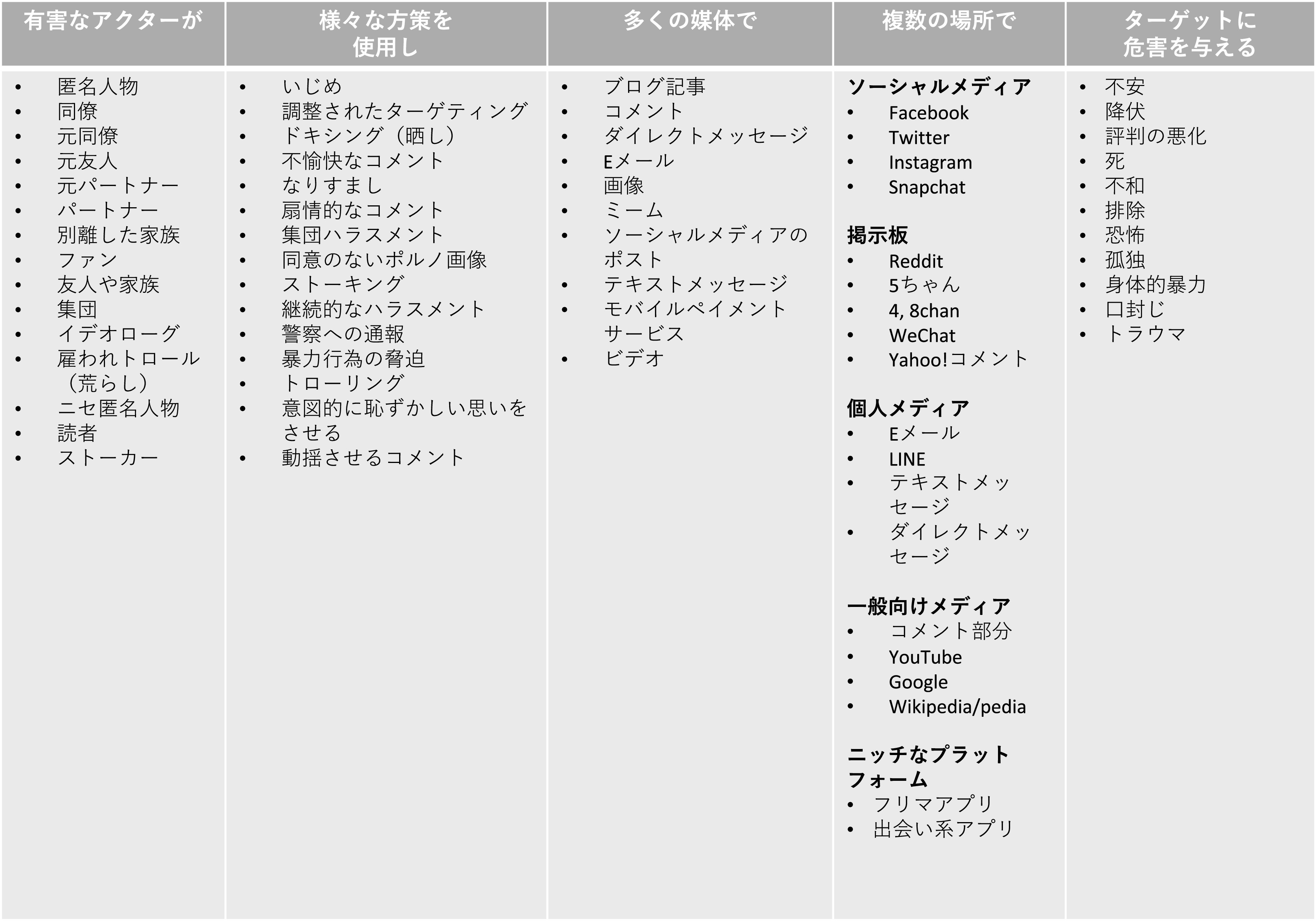
オンライン・ハラスメントの構成要素 ( Lee, 2019) に基づく.

こうしたオンライン・ハラスメントに対して、SNS プラットフォームはどのように対処しているのだろうか。ヤンコビッチら ( 2021) は、Facebook, Twitter, YouTube などユーザー数の多いSNSプラットフォームは、利用規約や「コミュニティ・ガイドライン」にハラスメント防止指針を取り入れているが、オンライン・ハラスメントの実態に比して画一的で、不十分であると指摘している。先述の PEN アメリカの調査においても、オンライン・ハラスメントを経験した 230 名のうち、53.1%がハラスメント被害をプラットフォームに通報したことがあると回答し、そのうち 70.8%がプラットフォーム側の対応は全く役に立たなかった、と回答している。

これらの既存研究は主に欧米の言語環境におけるオンライン・ハラスメントを分析したものであり、ハラスメントの定義や、ハラスメントを行った者および被害を受けた者もまちまちであるため、総花的であることは否めない。そこで本稿では、事例分析を通じて、日本語環境におけるオンライン・ハラスメントの被害とメカニズムの一例を詳述することを試みる。

## 事例分析 : X社の記者ツイート「炎上」事件

本稿では、質的アプローチを用い、事例分析を行った。研究パラダイムとしては、解釈主義に基づく。分析対象とした Twitter アカウントは、大手メディア企業 （以下X社とする） に所属する実名の女性記者のアカウント （以下Z氏）で、約 2 万のフォロワーを持ち、Twitter 社にも著名人であるという認証を受けている。筆者は、Twitter を使用し、従前より同アカウントをフォローしており、そのため同アカウントが以前に「炎上」の被害を受けていたことを確認していた。分析対象とした事例は、新型コロナウイルスによる死者数の多寡に関するツイートの掲載を発端とする炎上であるが、本ツイートがなされた時期は日本でも過去最多の新規感染者数が記録されていた頃であった （ 日本経済新聞, 2021a）。このことも関係してか、後述するとおり、本ツイートは多数のユーザーによって引用を含むリツイートがなされ、筆者らの目にも入ることとなったため、事例分析の対象として選択した。

Z 氏は、2021 年 8 月 3 日午後 7 時 12 分に、新型コロナ感染症拡大による死者の数について、これを少ないと表現するツイート等を見ると悲しくなる、数の多寡にかかわらず、亡くなった人一人ひとりに人生があり、それを「少ない」などと特に著名人が矮小化するのはいかがなものか、という内容のツイートをした。

本ツイートは掲載後、約 3 日間にわたり批判的なツイートが集中し、炎上状態となった。本稿では、2021年 8 月 3 日 〜7 日の間にZ氏アカウントに直接届いた 2,817 件の直接リプライ（直接リプライ1,175 件、これらのリツイート1,499 件、引用リツイート143 件）、及び 5,595 件の本ツイートの引用リツイート（以下引用 RT）の分析を行った。データ分析対象ツイートは、ExportData.io サービスを利用し、（to:[Z 氏のアカウントID]） until: 2021-08-07 since: 2021-08-03 および Query: https://twitter.com/[Z氏のアカウントID]/status/1422500544032038919, Result type: mixed, Language: ja で 2021 年 8 月 7 日に取得した。なお、同等の機能を果たす無料の代替品には、Twitter の公式 API ( https://developer.twitter.com/en/products/twitter-api、ないし https://developer.twitter.com/en/docs/twitter-api/tools-and-libraries) がある。

なお、直接リプライを中心に分析を行うのは、仮に引用RTが拡散されても、被引用ユーザーには通知が届かず、静観できる状況であるものの、被引用ユーザーに対して直接リプライ、もしくは引用 RT がなされると、本人に通知が届き、とりわけそのコメントが被引用ユーザー本人のアイデンティティ等、元ツイートの内容以外について批判的なコメントであった場合、ハラスメント行為に直面することとなり、精神的苦痛が大きいためである。

図 3 はZ氏アカウントへの直接リプライ及び直接リプライをリツイート、引用 RT したツイートの時間当たりの数をグラフ化したものである。これからは、一時間あたり直接リプライの数が、8 月 3 日午後 11 時台に一度ピークを迎え、低下した後、翌 8 月 4 日午後4時台から再び急増し、午後9時台になるまで、一時間あたり優に 100 近くのリプライが届く状態が継続したことが分かる。質的分析ソフト NVivo Windows （2020 年 3 月リリース版）を使用して、直接リプライの感情を自動で分類したところ、 図 4 のようになり、大部分の直接リプライが批判的な内容であることが判明した。なお同等の機能を果たすフリーの代替品として、 KH Coder 3 や、Python のライブラリ ML-Ask がある。これらを用いることで、ツイートに現れる単語の出現頻度や感情を分析することができる。

**図 3.**
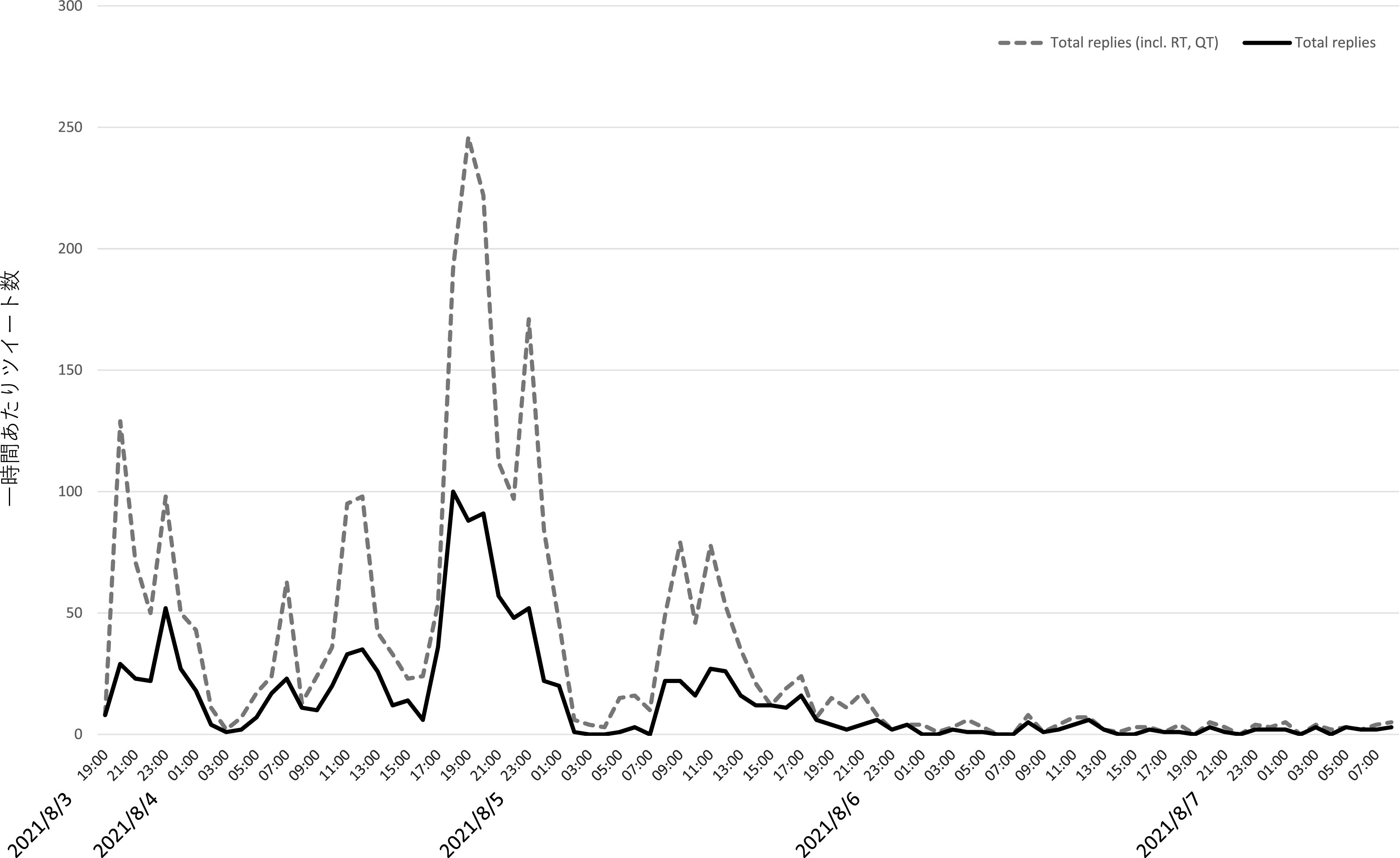
一時間あたり直接リプライおよびそれらのリツイート、引用 RT 数.

**図 4.**
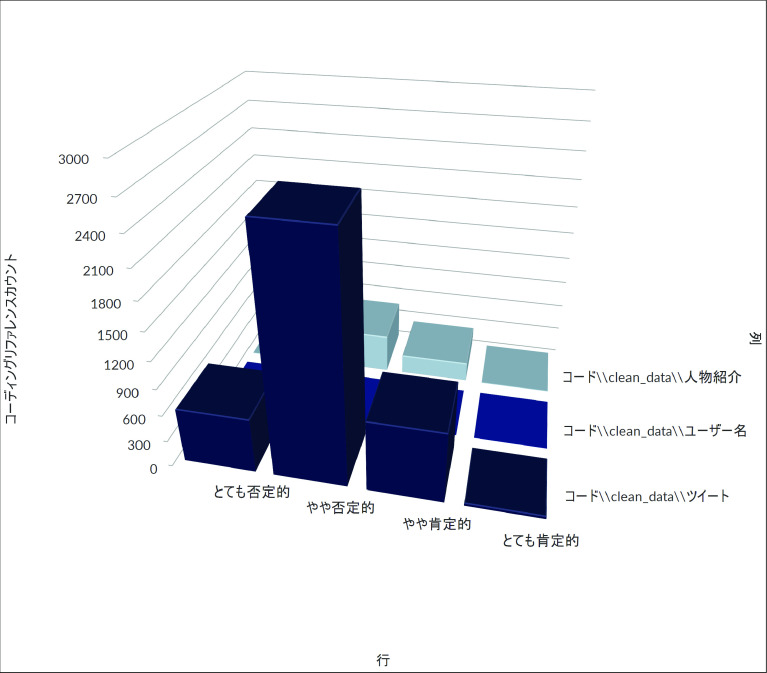
感情の自動コーディングの結果.

さらにツイートを手動で分類したところ、1,175 件の直接リプライのうち、肯定的な内容は 147 件、批判的な内容は 1,022 件となっていた。また、同ソフトを使用してワードクラウドを作成したものが、 図 5 である。図からは、直接リプライを送った者は「日本における （新型） コロナ （ウイルス感染症）による死者数は少ない」という旨を主張していることが分かる。

**図 5.**
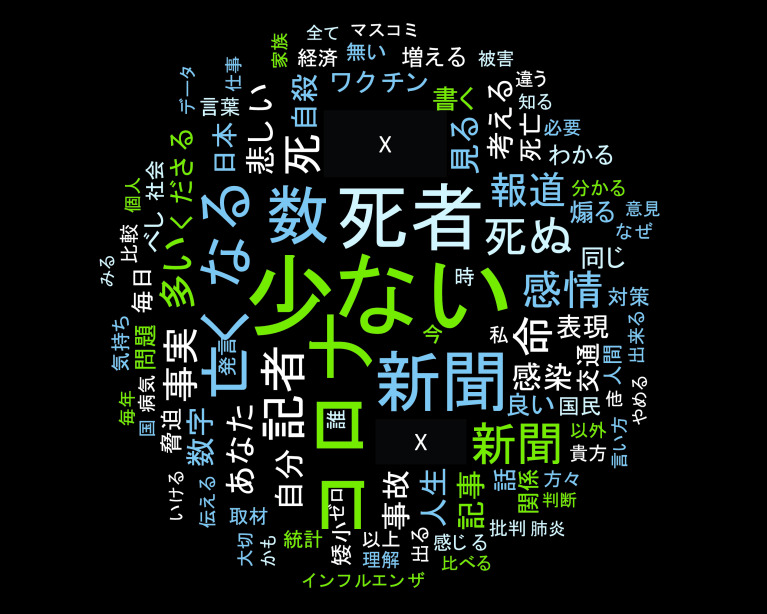
ワードクラウド (固有名詞は匿名化).

直接リプライを手動で分類したところ ^7^、 表 1 のとおりであった。最も数の多かった元ツイートの内容に対する主張は、「（死者数が）少ないという表現は妥当である」というもので（216 件）、次は「（新型コロナ感染症の）他にも重要な事柄がある」という内容のものであった（170 件）。これらは、形式的誤謬ないし偽善の抗弁である「そっちこそどうなんだ主義 (whataboutism) 」に基づくコメントである。また、71 件の直接リプライは、元ツイートが扇動的であると見なし、批判を行っていた。

**表 1.** 直接リプライの内容の分類と件数.

他にも重要な事柄がある		170
	COVID-19のみ特別扱いすべきではない	70
	経済困窮による死者	49
	HPVワクチン	10
	イスラエル	1
少ないという表現は妥当である		216
扇動的である		71
数字を「ちゃんと」報道すべき		30
女性差別的		150
	感情的になるな	124
	「ポエム」だ	16
政権批判		24
政権擁護		28
X社		186
マスコミ批判		177
仕方ない		39
個人攻撃		195

直接リプライは、元ツイートの内容ではなく、Z氏の属性に対して批判もしくは誹謗するものも多かった。例えば、「恥を知れ」「心の底から軽蔑する」「うるさい、クズ」「そんなことでよく記者をやっているものだ」といった、元ツイートの内容には全く関係のない個人攻撃が 195 件、X社批判が 186 件、マスコミ批判が 177 件、「感情的になるな」といった女性差別的なコメントが 150 件あった。なお、「女性が感情的である」というのはジェンダー・ステレオタイプ（「女は優しい」「男はたくましい」「女は世話好き」「男は数字ができる」のような、男女の性格や能力、役割、行動などに関する単純で固定化された考え方（ 森永, 2013））の一種で ( Weigard *et al.*, 2021)、こうした偏見が他者認知をゆがめ、性差別、なかでも善意的性差別 (benevolent sexism) が行われるとされる ( Glick & Fiske, 1996, 1997, 1999, Correll *et al.*, 2020)。日本語のオンライン環境においても、特に女性に対し「感情的である」として発話のパフォーマンスを攻撃することで、発言者の意見を抑え込むことが指摘されている（ 田中, 2020）。こうした理由から、「感情的である」というのは個人攻撃ではなく、女性差別的、すなわちZ氏が属する（とみなされている）集団に対する差別的発言であると判断した。

このように、Z氏がオンライン・ハラスメントを受けた理由としては、Z氏が政治的見解を述べたこと、個人的な意見を述べたこと、女性であること、外見 （ルッキズム）、という PEN アメリカの調査結果でも見られた、SNS 上で個人が意見表明を行ったこと、もしくは発言者のジェンダーやアイデンティティを理由に行われていることが分かる。これに加え、日本語インターネット空間独自のアイデンティティ攻撃の理由として、Z氏がX社の記者であるという側面があることが分かった。

次に、Z氏に対する直接リプライと、元ツイートの引用 RT の一時間毎の数量をグラフにし可視化、分析したところ、炎上を通じてハラスメントを行ったユーザーは、3 つの層に分かれることが推測された。すなわち、①インフルエンサー （高頻度炎上関与ユーザー群）、②インフルエンサーの「犬笛」に呼応する炎上加担ユーザー群、③荒らしを行うユーザー群、である。

小山ら (2019) による計約 13 万人の炎上参加ユーザーの分析によれば、炎上参加者は他の炎上にも参加しやすいことが明らかになっている。5 件以上の炎上に関わったユーザー77 名に関する詳細な分析においては、それらのユーザー間にはフォロー/フォロワー関係が密に存在し、高頻度炎上関与ユーザーは多くのフォロワー数を持ち、同じ炎上トピックに対してツイートを行うという、情報の共振構造が確認できたという。Z氏の炎上事件においても、多くのフォロワー数を持ち、特定の政治傾向を持つユーザーが、フォロー/フォロワー関係を結び、ユーザー間でZ氏のツイートの引用RTを順次行っていた（ 図 6, 図 7）。これらユーザー群が他の炎上に関わっていたかは本稿では確認できず、高頻度炎上関与ユーザー群とみなすことは難しいが、多くのフォロワー数を持つユーザーの引用 RT が、フォロワーを通じて広くTwitter 全般に拡散されていったことが推測できる（ 図 8）。

**図 6.**
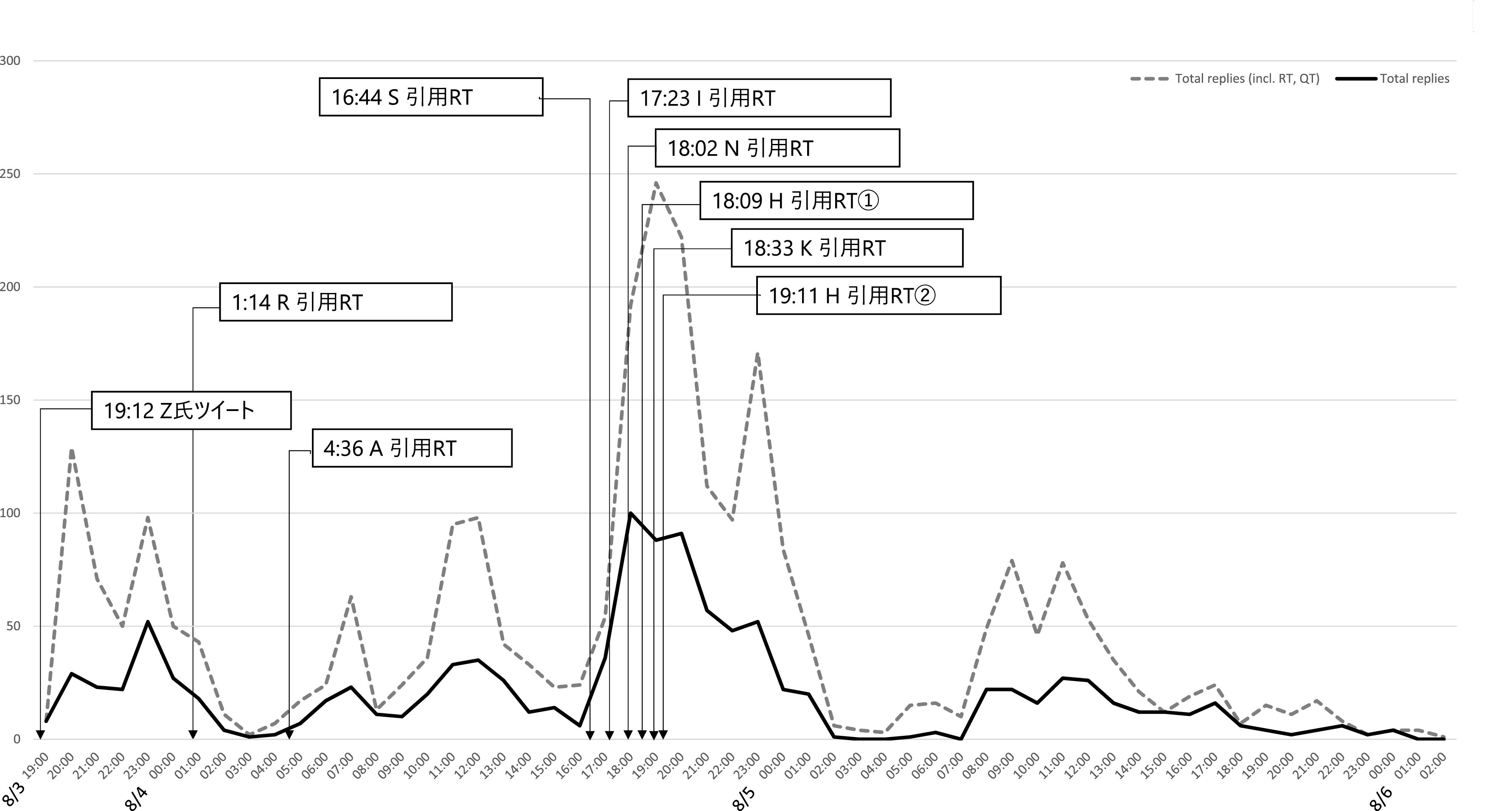
一時間あたり直接リプライとインフルエンサーの引用 RT.

**図 7.**
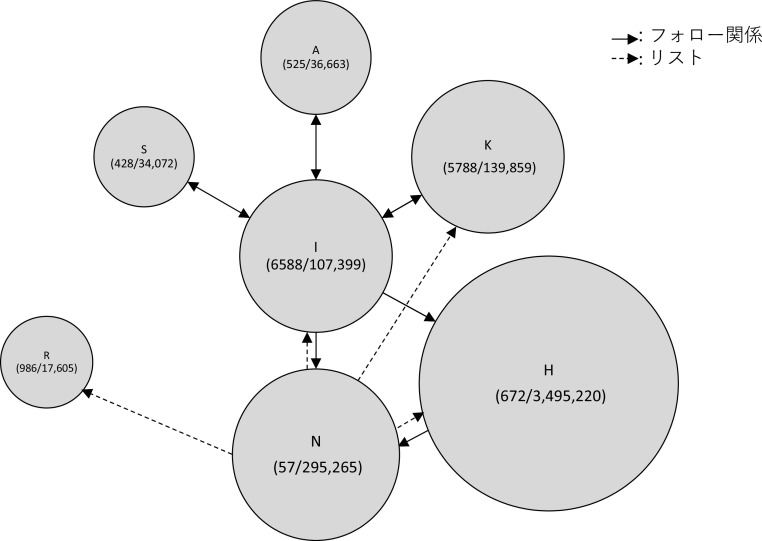
インフルエンサーのフォロー/フォロワー関係 (カッコ内フォロー数、フォロワー数).

**図 8.**
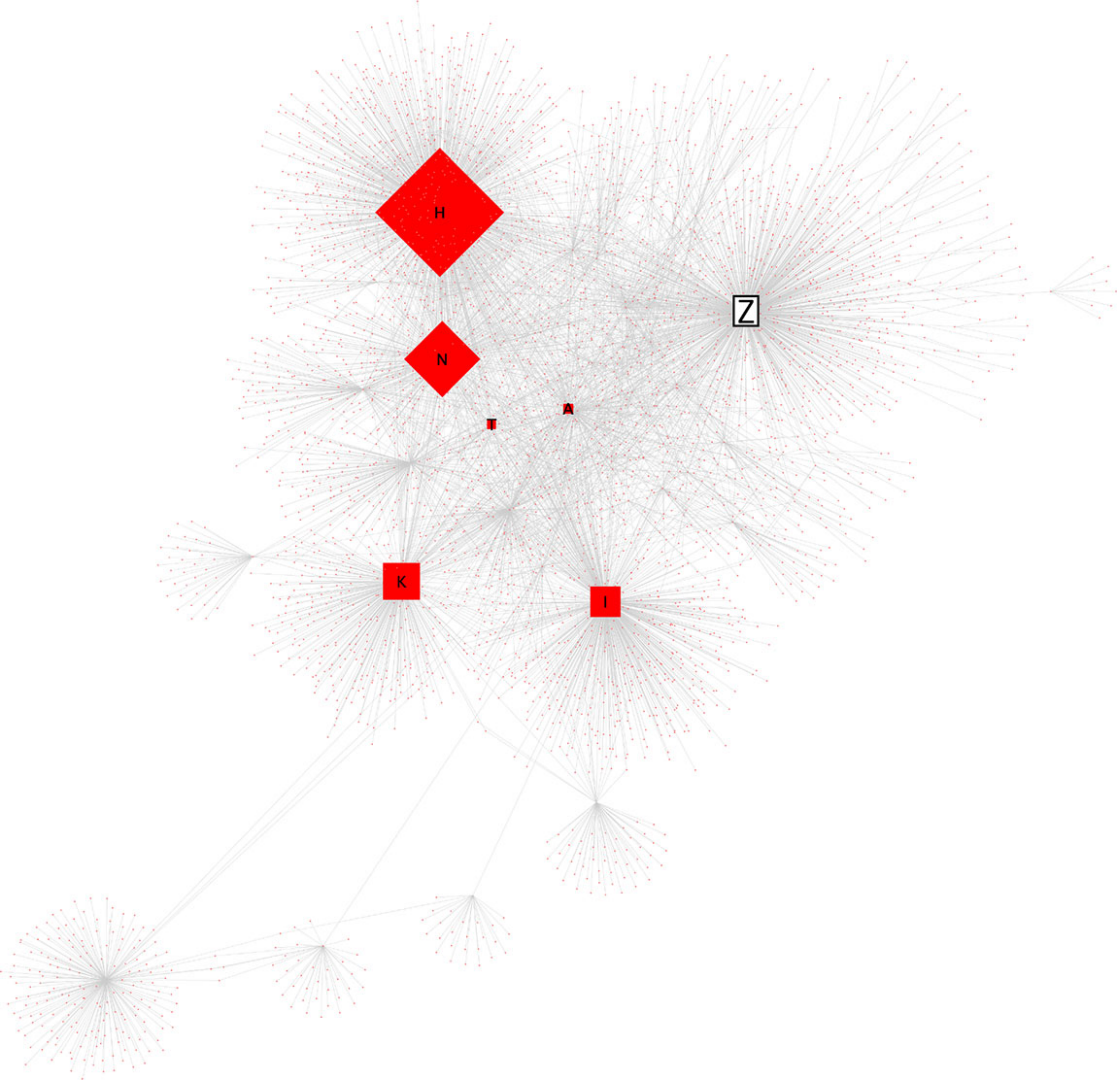
インフルエンサーによる引用RT拡散ネットワーク図.

②のインフルエンサーの「犬笛」に呼応するユーザー群であるが、犬笛とは「加害的もしくは有害な意味を持つ二重、もしくはコード化された言葉や記号を使い、インターネット上の加害者グループに特定の標的を攻撃するように合図する行為」を意味する ( PEN America, n.d.-a)。先のインフルエンサーのうち、約350 万ユーザーをフォロワーに持つH氏は、Z氏のツイートを2度に渡って引用 RT を行っている。第一回目の引用 RT は、2021 年 8 月 4 日午後 6 時 9 分に行われ、記者は新型コロナウイルスによる死者数は多いと読者の恐怖を煽ることで販売部数を稼ぐ最低のビジネスをしている、もっと科学を勉強して冷静かつ論理的に記事を書いてほしいが、無理であろうから当該メディア自体なくなってほしい、という内容であった。第二回目の引用 RT は、同日午後 7 時 11 分に行われ、Z 氏によるツイートは新型コロナウイルスに対する恐怖を煽って儲けることを正当化しており、最低である、という内容であった。

この 2 つのツイートに共通する主張が「煽るな」というものである。先述のとおり、直接リプライのうち、元ツイートが扇動的であると批判したものは71件であったが、これらの発生した時間帯を時系列で確認すると、それまでは合計 6 件しかこの内容の直接リプライがなかったものの、H 氏が一度目の引用 RT をした午後6時台は7件、二度目の引用 RT を行った午後7時台は 15 件、午後8時台は11件と、急増している（ 図 9）。また、H氏のツイートを見て来たと述べたツイートも3件あり、インフルエンサーの犬笛に呼応して、わざわざZ氏のアカウントに対して直接、故意の攻撃を行っていることが見て取れた。なお、 山口 (2015, 2016, 2020) による炎上現象の既存研究によれば、炎上加担の理由として正義感をあげる人が大半とあるが、本ユーザー群はこうした大多数の炎上加担者であることが推測される。

**図 9.**
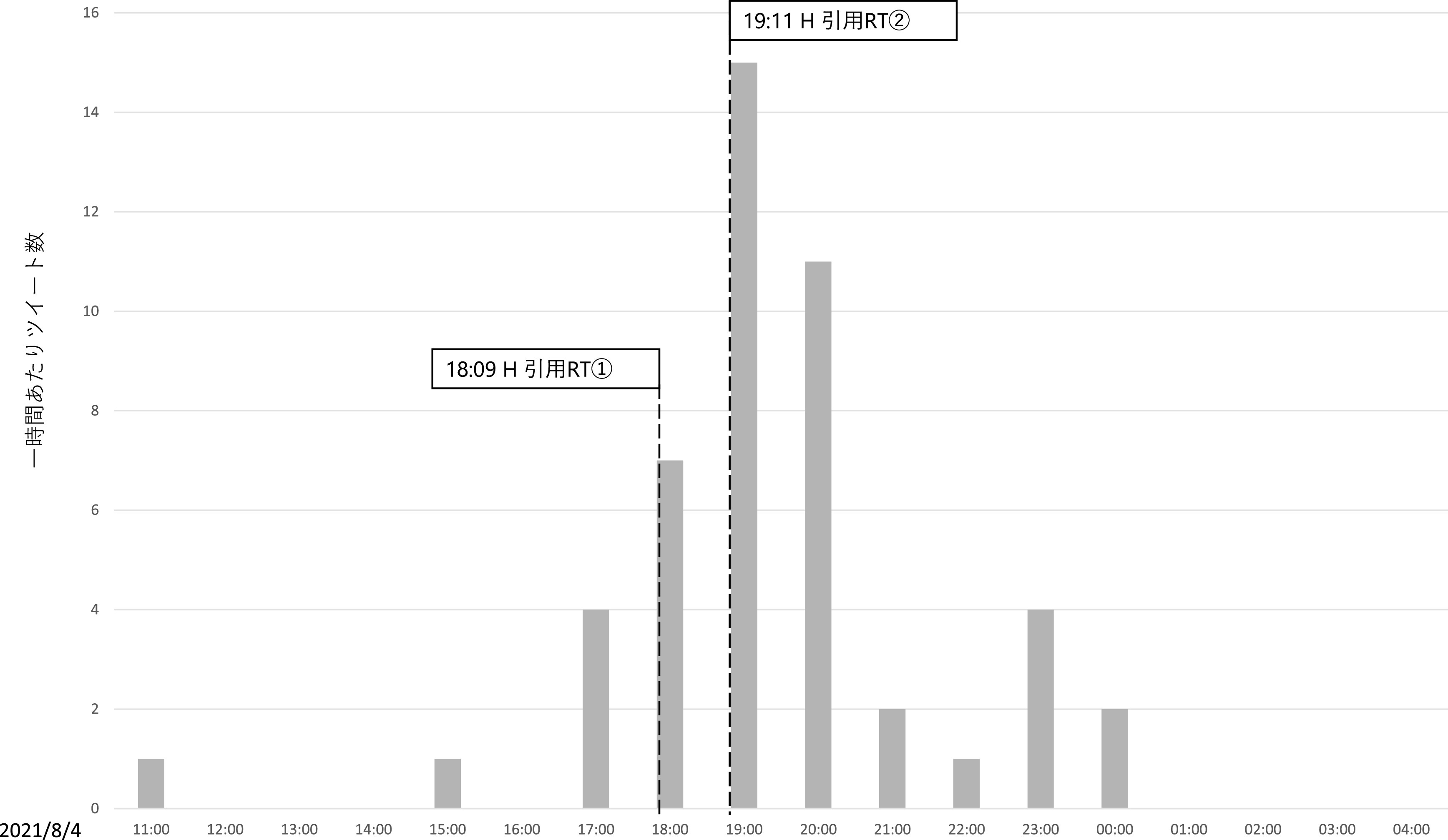
「煽るな」という内容の一時間あたり直接リプライおよびインフルエンサーの引用リツイート時間.

直接リプライの分析からは、インフルエンサー、及びインフルエンサーの呼びかけに直接・間接的に反応したと推測されるユーザーとは別に、③荒らし (扇情的なコメントやヘイトスピーチを繰り返し投稿すること)を行うユーザー群が見て取れた。まず、195 件の個人攻撃のツイートのうち、12 名のユーザーが 2 度以上、繰り返し個人攻撃のリプライをZ氏に送っていた。また、 図 10、 図 11 は批判的な内容の直接リプライ、及び個人攻撃のリプライの一時間あたりの数をグラフ化したものであるが、両者の数量は必ずしも呼応していない。特に一時間あたりの個人攻撃コメントが多かった時間帯（2021 年 8 月 4 日午前 11 時 〜8 月 5 日午前 2 時）について、先のインフルエンサーの犬笛に呼応した批判的ツイートの一時間あたり数と比較すると、特に増減傾向に類似性が見られない（ 図 12）。これらから推測されるのは、常日頃から Z 氏の発言をモニターし、期を見て荒らしを行うユーザー群が存在する可能性である。実際、自称メディアウォッチャーで7千余のフォロワーを持つユーザーが、2021 年 3 月 18 日に、X社には一部、低質かつイデオロギー色の強い記者がいる、という内容のツイートを行い、X 社の「低品質」記者一覧として、Z 氏ほか複数名の X 社に関係する記者の Twitter アカウント情報を掲載している。なお、こうしたユーザーが、Z 氏の他のツイートに対しても繰り返し荒らしを行っているか、またいかなる動機に基づいて荒らしを行っている・行ったのかはさらなる調査分析が必要である。 ^8^

**図 10.**
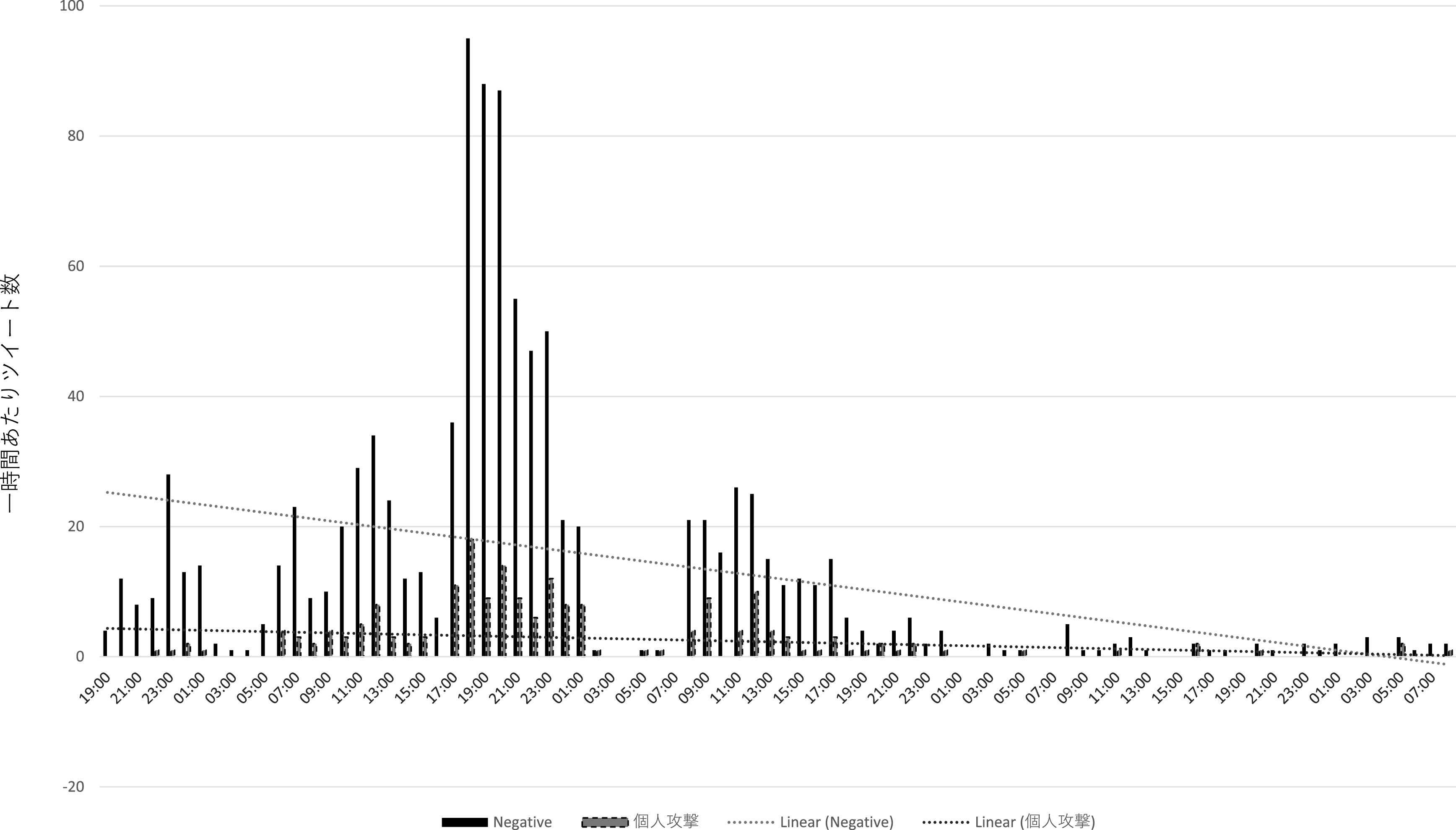
一時間あたり批判的コメントと個人攻撃コメント.

**図 11.**
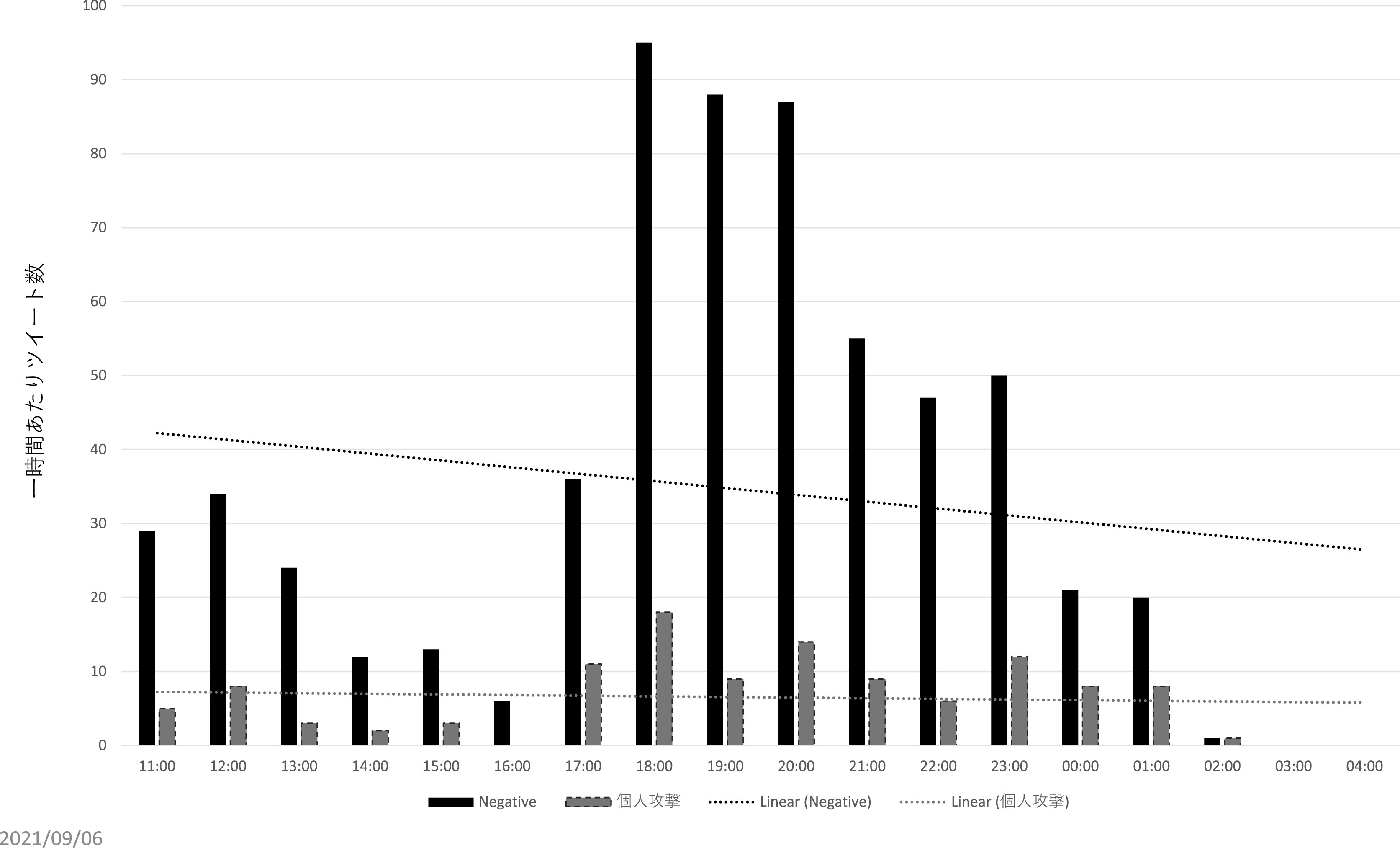
一時間あたり批判的コメントと個人攻撃コメント.

**図 12.**
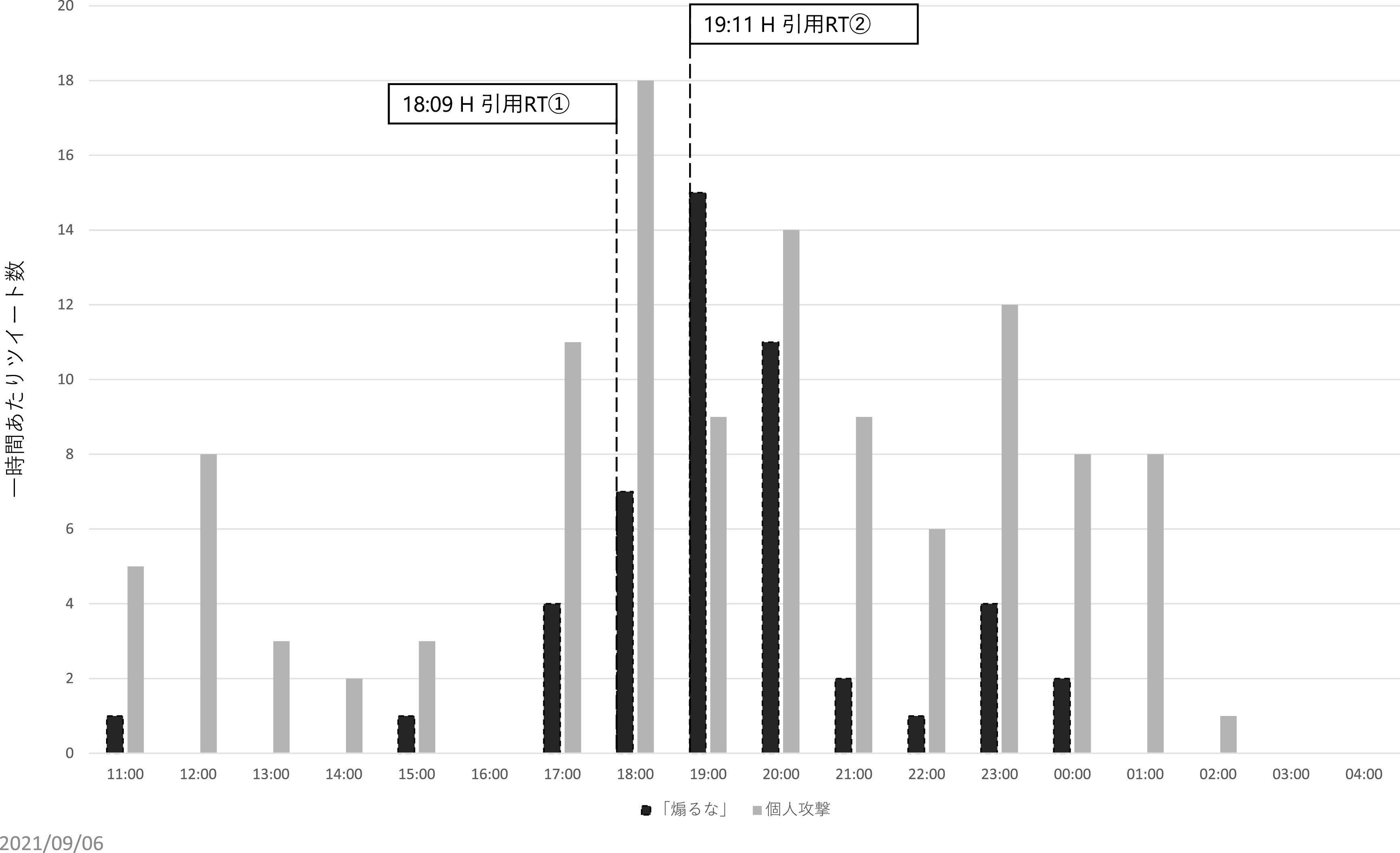
「煽るな」コメントと個人攻撃コメントの比較.

## オンライン・ハラスメント対策

国内外の既存研究から、誰であってもオンライン・ハラスメントを受ける可能性があること、一方でハラスメントを受けやすい属性があること、ハラスメントの被害は身体的・精神的に甚大であることが明らかとなった。また、日本語環境の Twitter における事例として、X 社の記者ツイート「炎上」事件を分析し、オンライン・ハラスメントを行うユーザーにはインフルエンサー群、インフルエンサーの犬笛に呼応する炎上加担ユーザー群、荒らしを行うユーザー群の三層があり、それぞれがなる形で一人のユーザーに対してハラスメントを行っていたことが見て取れた。このようなオンライン・ハラスメントに対して、ユーザーはどのような対策を取ることができるのだろうか。

各国の女性議員へのオンライン・ハラスメントの調査分析を行ったヤンコビッチら ( 2021) は、報告書に政策提言を記載している。具体的には、SNS プラットフォーム企業への提言として、

• 個別ツイートの通報ではなく、インシデント報告制度の導入
• プラットフォームから各企業・団体の SNS 担当者への情報共有
• 利用規約の適用の徹底
• ナッジ機能の強化
• プラットフォーム間のオンライン・ハラスメント防止機構の設置

政策担当者への提言として、

• SNS 規制の強化 （透明性のある報告システム、プラットフォームの報告義務）
• ディープフェイクなどに加え、ジェンダー化されたハラスメントも規制対象に含めるべき
• 米国で1994年に制定された「女性に対する暴力阻止法案」に、物理的な危害だけでなくオンライン上の危害も含めるべき ( Congress.gov, 1994)

雇用者への提言として、

• 被雇用者の SNS 発信に関するガイドラインの設定
• 心理的・道徳的・金銭的な支援。オンライン・ハラスメント被害者の晒し防止対策のためのサービス料の支払い、プラットフォームへの被害報告代行

を挙げている。しかし、日本語のインターネット環境の場合、オンライン・ハラスメントに関する社会的認知度が低い上に、主に外国に本社を置くプラットフォーム企業の対策も十分とはいえないため、ハラスメント防止策はユーザー個人、もしくは個人が所属する組織が取ることが求められているのが現状である。以下、日本語 SNS 環境の特殊性を念頭に置きつつ、個人および組織がとり得るオンライン・ハラスメント対策について、Twitter を中心に述べる。 ^9^

### オンライン・ハラスメント対策 (個人)

ハラスメント対策の最も基本的な行動としては、ミュート （特定のアカウントのツイートがタイムラインに表示されないようにする Twitter の機能）、ブロック （特定のアカウントによるメッセージの送付、ツイートの閲覧、フォローの禁止）、通報 （攻撃的なツイートまたはアカウントを Twitter 運営に報告すること）の 3 つがある ( PEN America, n.d.-d). また、いつどこでどのようなハラスメント被害を受けたのか、ログを取っておくことも重要である。こうした情報は、オンライン・ハラスメント行為を警察や弁護士に相談する際、重要な証拠となる（ 表 2）。

**表 2.** ハラスメント対応ログ.

日付	時間	詳細	結果・何をすべきか
			
			
			
			
			
			

また最近では、オンライン・ハラスメント対策の専用ツールが開発されており、こうしたツールの導入も間接的なハラスメント防止になり得る。例えば Block Party というツールは、誰からの返信が見たいか、見たくないかをフィルターとして設定することで、基準に該当する返信を全て自動でミュートする。これにより、ユーザー自身は Twitter を開いてすぐに嫌がらせのツイートを目にするというようなことを経験せずとも、自分自身で後日、もしくは信頼できる人物に依頼して返信の内容を確認することが可能になる ( Cho, n.d.)。さらに、Twitter 社が、「安全モード」機能を検討中である旨が発表された ( Doherty, 2021)。本機能を有効にすると、自分に対して有害な言葉を使ったユーザーや、荒らしを行ったユーザーが自動的に7日間ブロックされるという。その他、全般的なデジタル衛生のチェックや、SNSアカウントのチーム運用も対策として考えられる ( Jankowicz *et al.*, 2021)。

### オンライン・ハラスメント対策 (組織)

昨今は、議員、記者、研究者など既存メディアを通じて従来発信してきた者でなくても、SNS を通じて発信し、広く社会にアウトリーチすることが求められている。またZ氏の例では、Z 氏がX社に所属することを理由にハラスメントが行われていた。炎上やオンライン・ハラスメントは個人アカウントのみならず、大小様々な組織に対しても発生している（ 榊・鳥海, 2020）。したがって、所属員の SNS アカウント運用とそれに伴うハラスメント被害について、組織が全く関与しないことは難しい。

組織ができる対策として、まずは炎上決着メカニズムを理解することが不可欠である。田代・折田 ( 2012) は、ネット炎上を「不具合」に対して決着をつけようとすること、と見なし、炎上が収束するためには「決着」が必要とした。そして、ハラスメントが収束するためには攻撃側が納得することが必要であり、法的に問題があれば法的に処理されることで収束に向かう（ 図 13）。謝罪を行ったとしても、単に謝罪しただけでは収束せず、謝罪が受け入れられて収束する。炎上が議論へ繋がって収束する、コメント欄やブログそのものの削除 (Twitter の場合はリプライ機能の制限や Twitter アカウントの削除)、炎上を無視し、忘れ去られることで収束を迎えることもある、とする。所属員の SNS アカウントが万が一炎上したり、ハラスメントの被害者となった場合、そうした行為を受けたことを批判したり懲罰の対象とするのではなく、収束には様々なパターンがあることを理解することが、SNS 運用者支援の第一歩であろう。本稿は質的研究であり、実証や仮説検証を目的とするものではないこと、また本稿の問いは「どうなっているか?」という記述的問いであり、「どうすればいいか?」という「処方的問い」ではないことから（ 大谷, 2019）、本稿の分析から得られた知見を対策の一助とすることには慎重でありたい。他方、前節で扱った事例から、オンライン・ハラスメントを行うユーザーにはいくつかの層があり、それぞれが異なった形でハラスメントを行っていたことが観察できていたことを念頭に置いて、炎上やハラスメントの現象を解釈することも有用であるかもしれない。

**図 13.**
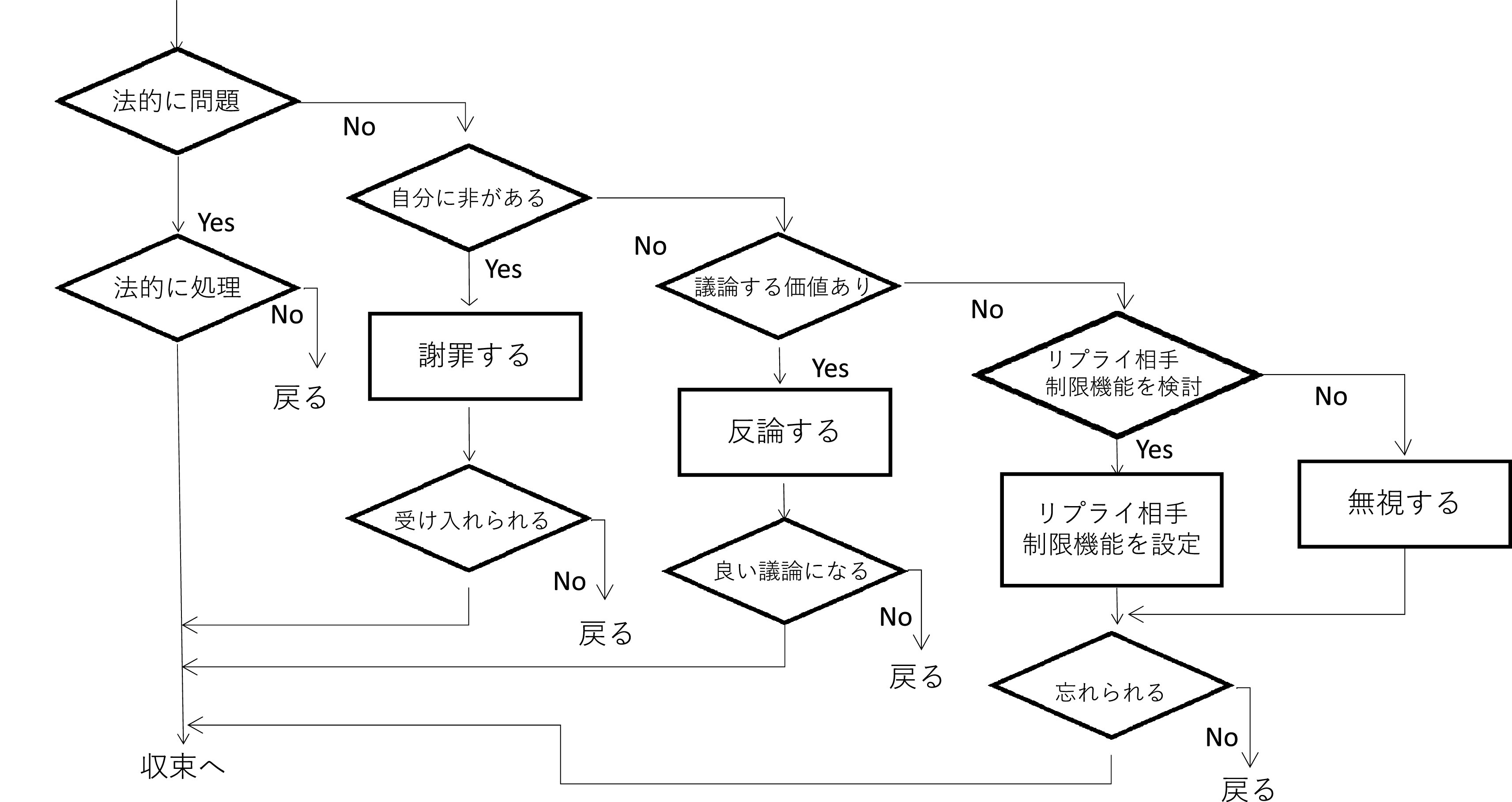
決着のフロー (Twitter を想定、 田代・折田 2012) に基づく.

第二に、組織がインターネット上でどのような属性を与えられているか、それに基づく荒らしユーザーの確認が求められる。事例分析ではメディア企業である X 社の例を扱ったが、他にも例えば大手消費財化学メーカーである花王は、韓流ドラマをよく流すフジテレビへのスポンサーを多くつとめていたことから、「韓国に貢献し日本文化を破壊する企業である」という誤情報が拡散し、インターネット上で批判を受けた （ 田代・折田, 2012）。先述のとおり、オンライン・ハラスメントには加害者の標的になりやすい属性の集合があり、集合の共通部分においては最もハラスメントに遭う確率が高くなる。企業が自社のブランド認知度を調査するのと同様、ハラスメントの標的となる可能性についても検討する必要があるだろう。

先に、個人ではミュート、ブロック、通報が最も基本的かつ効果的なハラスメント対策であると述べた。他方で、議員、記者、公的組織の所属員によるアカウントなど、発信する情報に公共性があると見なされる場合、ブロック行為が好ましくないと見なされるケースもある （ 日本経済新聞, 2021b）。したがって、組織内でブロック機能の利活用について議論の上、ガイドラインを設け、適切に運用することが求められる。仮に所属員が自己防衛としてとり得る有効策を、所属組織が禁じるようであれば、所属員がハラスメントを受け身体的・精神的ダメージを受ける可能性も高まるのであるから、より充実した心理的・道徳的・金銭的な支援が必要となる。

日本では、2021 年 4 月 21 日、インターネット上で誹謗中傷の投稿を行った人を特定しやすくするためのプロバイダー責任制限法の改正案が可決され、加害者の特定にかかる手続きが簡素化された。また、10 月 7 日には、刑法の「侮辱罪」に懲役刑を追加する法改正の要綱案が、法制審議会の部会により取りまとめられ、法務大臣に答申するとの報道がなされている （ 共同通信, 2021）。それでも開示手続きなど、オンライン・ハラスメントの直接的・間接的費用の負担は加害者よりも被害者にとって重いことは変わらない。したがって、例えば、弁護士保険の費用一部負担といった形で、組織が所属員への金銭的支援を行うことを検討すべきであろう。

## おわりに

国内外の先行研究から、誰であってもオンライン・ハラスメントを受ける可能性がある一方で、ハラスメントを受けやすい属性があること、また、ハラスメントはオンラインで行われるものであっても、被害者が受ける身体的・精神的ダメージは看過できないことが明らかとなった。日本語環境の Twitter におけるオンライン・ハラスメントの事例として、大手メディア企業X社の記者ツイート「炎上」事件を分析した結果、ハラスメントを行うユーザーにはインフルエンサー群、インフルエンサーの犬笛に呼応する炎上加担ユーザー群、荒らしを行うユーザー群の三層があること、さらに、それぞれが異なる形で一人のユーザーに対してハラスメントを行っていたことが観察できた。事例分析は炎上事例の一例でしかないため、一般化は難しいが、SNS 上の炎上現象に関する先行研究の結論を補完するものである。本稿で導出した仮定に基づき、より大規模な分析を行うことを将来の課題としたい。

本稿の最後には、日本語のインターネット環境の場合、オンライン・ハラスメントに関する社会的認知度が低いことや、Twitter など外国に本社を置くプラットフォーム企業に対策を求めることの難しさを鑑み、SNS ユーザー個人、もしくは個人が所属する組織が取るべきハラスメント防止策を詳述した。日本語環境の SNS については、オンライン・ハラスメントという概念自体が知られていない状況であり、その社会的影響に比して、ハラスメントにあった被害者を中心に検討した論考が少ない。こうした意味で、本稿が学術的な議論の広がりと深化の一助となり、実効的なハラスメント防止策に繋がることを期待したい。

### データ利用可能性

本論文の研究結果の基礎となるデータは，すべて本論文中に示されており，追加のソースデータは必要とされていない。例外として、ソーシャルメディアのデータには倫理上および著作権上の制限があるため、本研究の基礎データを共有することはできない。対象となるデータは、2021 年 8 月 3 日に投稿された大手メディア企業に所属する女性記者（以下Z氏）のツイートと 2021 年 8 月 3 日 ~7 日の間に Z 氏アカウントに直接届いた 2,817 件の直接リプライ（直接リプライ1,175 件、これらのリツイート1,499 件、引用リツイート143 件）、及び 5,595 件の本リツイートの引用リツイートである。本研究の再現を可能にする詳細な情報は、「事例分析」部分に記載されている。方法に関する質問がある場合は、著者 (tonami.aki.ka@u.tsukuba.ac.jp) に連絡されたい。

Tweet ID についても、Z 氏の匿名性を守るためのセキュリティ上の配慮から共有しない。同分野の研究者が、善意と明確な研究目的をもってIDの閲覧を希望する場合、データの利用目的と方法を明記の上、著者 (tonami.aki.ka@u.tsukuba.ac.jp) に連絡されたい。

### 謝辞

本稿の執筆にあたり、M氏には荒らしユーザーや個人でできるオンライン・ハラスメント対策について助言をいただいた。また、査読者の方々からも大変有用なコメントをいただいた。心から感謝申し上げます。

## Footnotes

^1^
個人、コミュニティ、組織が、簡単にアクセスできるユーザー生成コンテンツを作成、共同作成、修正、共有、関与することを可能にすることで、共同作業、接続、相互作用、コミュニティの構築を可能にする Web ベースのサービスのこと（ McCay-Peet & Quan-Haase, 2017）。

^2^
情報操作型のサイバー攻撃のこと（ 長迫, 2021）。

^3^
同調査では、「根拠のない悪口を言いふらして他者の名誉を傷つけたり、人格を否定するような言葉で他者を傷つけること」と定義されている。

^4^
その他、類似の用語として、ネット上いじめ、サイバーブリングなどがあるが、執筆時点では、それぞれの用語の定義は確定していないように見受けられる。今後のさらなる議論と整理が望まれる。

^5^
2014 年夏頃から欧米で発生した、表現の自由を掲げるゲーム愛好者と、女性差別の観点から表現規制を求めるフェミニストらの対立がインターネット上で激化した事件。特定の女性プログラマーに対し、個人情報の流出、殺害予告、脅迫を含む過激なオンライン・ハラスメントが行われた。

^6^
図2では、オンライン・ハラスメントが Twitter 以外の複数のプラットフォームに渡り行われていることが指摘されている。こうした行為の分析は本稿の分析対象範囲を越えるため、今後の研究課題としたい。

^7^
同一ツイートに複数のコードが含まれる場合もある。本稿ではデータを読みながら探索的に自由にコードを付す「生成的コーディング generative coding」を行った。質的研究で採用されることの多いこのコーディングでは、解釈が重要な機能を果たし、主体的なものと考えられる。このため、複数のコーディング作業者をおくことは稀で、選定されたコード群からコードを選んで付し、複数のコーディング作業者を置いてその間の一致度を評価する「標準化コーディング standardized coding」とは異なる（ 大谷 2019）。

^8^

インターネット上の緩いネットワークでつながる、荒らし（トロール）コミュニティの集団行動の原理については、 エブナー (2021) に詳しい。荒らしはインターネット黎明期から存在していたが、2016 年の米国大統領選以降、荒らし行為のかたちをとった政治戦争が新常態になり、特に女性のアーティスト、活動家、政治家を標的としているという(p.162, p.181)。

^9^
本稿では Twitter で行われたハラスメントを分析の中心に置いたが、その他の SNS におけるハラスメント対策の検討は今後の課題としたい。
