# Supplementary material for: Online harassment in Japan: Dissecting the targeting of a female journalist
Source: F1000Res. 2022 Feb 3;10:1164. [Article in Japanese] Originally published 2021 Nov 17. [Version 2] doi: 10.12688/f1000research.74657.2 (PMC8861472; doi:10.12688/f1000research.74657.2)
Supplement: Peer review history

# Peer review: Reviewer response for version 2

**DOI:** 10.5256/f1000research.120282.r122066

**Published:** 2022-02-21

**Authors:** Shinichi Yamaguchi

**Affiliations:** 1 Center for Global Communications, International University of Japan, Niigata, Japan

　投稿時から社会的意義の高い優れた論文でしたが、さらに先の査読コメントに対して、全て適切な修正と反論がなされました。先行研究レビューの充実、Research Questionの明示、手法の解説、研究課題の明示など様々な点で改善が見られ、非常に完成度の高い論文になったかと考えます。

 　オンラインハラスメントは多くの被害者が存在し、重要なイシューであるにもかかわらず、とりわけ国内の研究は少ないのが現状です。オンラインハラスメントの実態と対策を示す本論文が、多くの個人・組織を救うと共に、サービスの改善や適切なルール作りに寄与することを期待します。

 　一方で、筆者らやもう1人の査読者である川畑先生がご指摘されているように、研究課題もいくつか存在し、様々な観点からまだまだ広がりのあり得るテーマだと思います。研究の今後の発展を大いに楽しみにしています。

（該当する場合は要回答）統計分析および解釈は適切ですか。

一部該当

本研究は明確かつ正確に提示されたものであり、最新の文献を引用していますか。

はい

方法と分析について第三者による再現が可能となるよう十分な詳細が提示されていますか。

一部該当

結果の基礎となるソースデータはすべて入手可能で再現性を十全に保証していますか。

ソースデータは不要

結論は結果により妥当な裏付けを得ていますか。

一部該当

研究設計は適切で学術的価値がありますか。

一部該当

Reviewer Expertise:

計量経済学, ソーシャルメディア, 社会情報学, 情報経済論

I confirm that I have read this submission and believe that I have an appropriate level of expertise to confirm that it is of an acceptable scientific standard.

# Peer review: Reviewer response for version 1

**DOI:** 10.5256/f1000research.78433.r100521

**Published:** 2021-12-20

**Authors:** Shinichi Yamaguchi

**Affiliations:** 1 Center for Global Communications, International University of Japan, Niigata, Japan

本研究では、オンライン・ハラスメントという概念を導入し、その概要を明らかにすると共に、Twitterで発生した炎上事例を分析してハラスメントを行うユーザを整理しています。また、最後には個人と組織がとるべき対策について詳細に述べられています。著者らが指摘するように、炎上の被害に遭った当事者視点に基づいて分析した研究は少なく、そこにオンライン・ハラスメントという概念を導入して整理・分析を行ったことは意義が高いといえます。実証分析も丁寧で、社会の関心も高い分野といえ、研究成果に関心のある読者も多いでしょう。

 　しかしながら、これを学術論文として考えた時に、いくつか問題点を抱えていると思います。具体的には、以下の点に改善出来るところがあると思います。

①既存研究レビュー・分析・対策の関連性がない

 　最も大きいのが、各節の関連性がなく、報告書のように知見を羅列しているところだと思います（各知見自体は非常に有意義なものですが、学術論文としては課題があるという意味です）。例えば、大きく紙幅を割いている図2について、「悪意をもつ行為者が、様々な方策を使用し、多数の媒体を通じて、複数の場所で、標的（被害者）に危害を与えるメカニズムが存在する」と指摘している一方で、分析されているのはTwitterのみです。さらに、結構な分量を割いている「オンライン・ハラスメント対策」も実証分析と全く関係のない記述が延々と続いています。せっかくハラスメントユーザのパターンを明らかにしたのに、活かされていません。

 　その要因の1つに、節の構成があると思います。まず、序盤では「はじめに」と「オンライン・ハラスメントの現状」とあります。後者は既存研究レビューといえますが、本当に現状を述べるのが中心となっており、既存研究にはどのような課題があり、そのために本研究では～を明らかにするという明確な研究の問いが示されていません。強いて言うなら「欧米の言語環境の研究しかないので日本語環境での一例を記述する」というところがそれに該当すると思いますが、もう少し本研究の新規性・社会的意義を示すためにも、既存研究との位置づけとResearch Questionをはっきりさせた方が良いと思います。そのためには、既存研究よりも、本実証分析の内容に関連するものが必要だと思います。具体的にいうと、オンライン・ハラスメントの攻撃者を分析した研究です。Research gapやResearch Questionについて、別途節を設けても良いかもしれません。

 　また、事例分析の節では手法と分析結果が一緒の節に入っているので、分けて書くと同時に、手法をもっと詳しく書いたほうが良いと思います。特に、本研究で重要な知見である3つの層の分類について、「元ツイートの引用RTの関係を分析」としか書かれておらず、手法が分かりませんでした。

 　そして、得られた結果と先行研究との関係を議論する節や、結果から導かれる対策を論じる結論節を用意したほうが良いと思います。現在の「オンライン・ハラスメント対策」で書かれている内容は興味深いのですが、これらは縮小させ、分析から得られる対策の検討に紙幅を割くことを推奨します。

②事例が1つしかない

 　1事例をもとにオンライン・ハラスメントのパターン分析をするのは、やや無理があるように思いました。事例分析なので数は多くなくて良いと思いますが、頑健性を検証するためにももう2，3事例の分析があった方が良いと思います。ただし、これは難しいと思うので、修正要求ではなく修正提案となります。

③その他（細かい点）

 ・分析において、「「感情的になるな」といった女性差別的なコメント」とありますが、「感情的になるな」単体で女性差別的と解釈してよいのか疑問が残りました。女性差別的に使われることが多いのは理解していますが、男性相手にも使う言葉だからです。また、「心の底から軽蔑する」が個人攻撃に入るのならば、「感情的になるな」も個人攻撃なようにも思います。

 ・小山（2019）が文献リストに見つかりませんでした。

（該当する場合は要回答）統計分析および解釈は適切ですか。

一部該当

本研究は明確かつ正確に提示されたものであり、最新の文献を引用していますか。

はい

方法と分析について第三者による再現が可能となるよう十分な詳細が提示されていますか。

一部該当

結果の基礎となるソースデータはすべて入手可能で再現性を十全に保証していますか。

ソースデータは不要

結論は結果により妥当な裏付けを得ていますか。

一部該当

研究設計は適切で学術的価値がありますか。

一部該当

Reviewer Expertise:

計量経済学, ソーシャルメディア, 社会情報学, 情報経済論

I confirm that I have read this submission and believe that I have an appropriate level of expertise to confirm that it is of an acceptable scientific standard, however I have significant reservations, as outlined above.

# Response

**Published:** 2022-01-13

**Authors:** Aki Tonami

**Affiliations:** University of Tsukuba, Japan

山口先生

 査読コメントをいただき、誠にありがとうございます。

①既存研究レ ビュー ・分析・対策の関連性がない

- 本ジャーナルの研究論文の前提として「Research Articles should present originality in findings and insights and offer theoretical, empirical, experimental and/or methodological advances to their respective fields of research. Null and negative findings and reanalyses of previous studies leading to new results, as well as confirmatory results, are also encouraged.」とあります（ https://f1000research.com/for-authors/article-guidelines）。研究の独自性を提示するに際し、どの程度先行研究のレビューに紙幅を割くべきかは分野によって異なりますが、本稿ではあえて、オンラインハラスメントが言語、文化、文脈に高度に依存することから、主に英語による、英語圏におけるオンラインハラスメントに関する先行研究の網羅的レビューを避け、そもそも存在していない日本語環境におけるオンラインハラスメントの論考の第一号となることを目指しました。したがって、Research gapは既存研究がないこと、となります。また本稿は質的研究であり、実証や仮説検証を目的とするものではありません。よって、Research questionは「オンライン・ハラスメントの現状はどうなっているか」という記述的問いになります。ただしこれが分かりにくいとのご指摘かと存じますので、その旨加筆修正いたしました。
- なお、本論文が発表された後に発表されたオンラインハラスメントに関する日本語の研究では、例えば、法学セミナー2021年12月号特集『言論に対するゆるしと制裁』が参考になるかと考えます。
- 「学術論文としては課題がある」とのご指摘については、想定されている学術論文が具体的にどのような論文なのか不明なため、前提と求められている条件を明示していただければ幸いです。（例えばIMRaDの形式で書かれることの多い量的研究の論文なのか、など。）
- 図2において、オンラインハラスメントがTwitter以外の複数のプラットフォームに渡って行われていると述べているのはご指摘のとおりです。しかしながら、マルチプラットフォームにおけるハラスメントについては本稿の対象範囲外ですので、今後の課題として脚注を追加いたしました。
- 手法を「もっと詳しく書いたほうが良い」とのこと、「元ツイートの引用RTの関係を分析」について詳細を記述しました。もし、その他にも不足点がある場合、何をどの程度詳細に書けば、いずれの基準にもとづいて適切と思われるのか、ご教授いただければと思います。

②コメント「事例が 1 つしかない」

 論文において本事例をもってのみ一般化することは難しく、SNS上の炎上現象に関する先行研究の結論を補完するものである、と記述したとおりです。

③その他（細かい点）について

- 第一のコメントは質的データ分析を行った際のコーディングについてのご質問かと思いますが、「女性が感情的である」というのはジェンダー・ステレオタイプ（「女は優しい」「男はたくましい」「女は世話好き」「男は数字ができる」のような、男女の性格や能力、役割、行動などに関する単純で固定化された考え方（森永 2013））のひとつで（Weigard et al. 2021）、こうした偏見が他者認知をゆがめ、性差別、なかでも善意的性差別（benevolent sexism）に繋がることは既存研究で明らかになっています（Glick & Fiske, 1996, 1997, 1999, Correll et al., 2020）日本語のオンライン環境においても、特に女性に対し「感情的である」として発話のパフォーマンスを攻撃することで、発言者の意見を抑え込むことが指摘されています（田中, 2020）。男性も「感情的になるな」と批判を受けることがあるとのご指摘ですが、これは「感情的になること」――前述のとおり、一般的に女性の性格や能力、役割、行動に紐付けられているステレオタイプですが――が誤っている、よくない点であると捉えられているため、指摘しあげつらわれていると考えられます。こうした理由から、「感情的である」というのは個人攻撃ではなく、女性差別的、すなわち対象者が属する（とみなされている）集団に対する差別的発言であると判断しました。
- 他方、個人攻撃については、対象者が属する集団（この場合、女性という性グループや、メディア企業）ではなく、記者本人に対する攻撃をこのカテゴリーに分類しました。著作権とプライバシーの保護に留意しつつ、それと分かりやすい例を追加いたしました。
- なおコーディングについては、本稿ではデータを読みながら探索的に自由にコードを付す「生成的コーディング generative coding」を行いました。質的研究で採用されることの多いこのコーディングでは、解釈が重要な機能を果たし、主体的なものと考えられます。また、このため複数のコーディング作業者をおくことは稀で、この点は選定されたコード群からコードを選んで付し、複数のコーディング作業者を置いてその間の一致度を評価する「標準化コーディング standardized coding」とは異なることを申し添えます（大谷 2019）。
- オンラインハラスメント対策に関しては、先述のとおり、本稿は質的研究であり、実証や仮説検証を目的とするものではないこと、またResearch questionも「どうなっているか？」という記述的問いであり、「どうすればいいか？」という「処方的問い」ではないことから（大谷 2019）、本稿の分析から得られた知見を対策の一助とすることは回避しました。他方、主に英語圏の先行研究、及び「炎上」についての日本語の先行研究には対策案として採用されるべき貴重な知見も示されており、それらを日本語のインターネット環境や、個人・個人が所属する組織の実務的な観点から検討し、有用と思われるものを提言いたしました。こうした背景が分かりにくいことはご指摘のとおりと考えますので、加筆修正いたしました。
- 小山ら（2019）の論文の文献リスト漏れについてご指摘ありがとうございました。その他の追加論文とともに挿入しました。

参考文献

 Correll SJ, Weisshaar KR, Wynn AT, *et al.*: Inside the Black Box of Organizational Life: The Gendered Language of Performance Assessment. *Am. Sociol. Rev.* 2020; 85(6): 1022–1050. https://doi.org/10.1177/0003122420962080

 Glick P, Fiske ST: The Ambivalent Sexism Inventory: Differentiating Hostile and Benevolent Sexism. *J. Pers. Soc. Psychol.* 1996; 70: 491–512. https://doi.org/10.1037/0022-3514.70.3.491

 Glick P, Fiske ST: Hostile and Benevolent Sexism. *Psychol. Women Q.* 1997;

 21(1): 119–135. https://doi.org/10.1111/j.1471-6402.1997.tb00104.x

 Glick P, Fiske ST: The Ambivalence toward Men Inventory: Differentiating hostile and benevolent beliefs about men. *Psychol. Women Q.* 1999; 23(3): 519–536. https://doi.org/10.1111/j.1471-6402.1999.tb00379.x

 Weigard A, Loviska AM, Beltz A: Little evidence for sex or ovarian hormone influences on affective variability. *Scientific Reports*. 2021; 11, 20925. https://doi.org/10.1038/s41598-021-00143-7

 大谷 尚: 質的研究の考え方　研究方法論からSCATによる分析まで. 名古屋大学出版会; 2019.

 田中 東子: なぜ男性より女性のほうがインターネットで炎上しやすいのか　「女性らしくない女性を罰したい」.2020. (accessed 22 December 2021). Reference Source

 小山 耕平, 浅谷 公威, 榊 剛史, *et al.*:ネット炎上におけるユーザーの共振構造. *The 33rd Annual Conference of the Japanese Society for Artificial Intelligence.*

 2019.

# Peer review: Reviewer response for version 1

**DOI:** 10.5256/f1000research.78433.r100519

**Published:** 2021-12-09

**Authors:** Yasuko Kawahata

**Affiliations:** 1 College of Social Relations, Department of Communication and Media Studies, Rikkyo University, Tokyo, Japan

イントロダクション・前文では、オンライン・ハラスメントに関しての発生起因における最新の動向に関する研究、事例紹介などの論考が掲載されており、一般の方々を対象とした昨今のオンライン上におけるリスクマネジメントに関する議論がまとめられているコレクティブ・インフォメーションとしての役割を果たすだろうと考えました。

 （1）全体のレビュー

 一方で、今回の記事はジャーナリストを対象にした比較的顕著な一例における事例であるかとも存じます。

 そういった面では、多くの課題が示唆できる論文でもあり、今後も議論が続くであろうオンライン・ハラスメントにおける問題解決の活路を提示する位置づけの論文としての役割も果たしていると考えております。今回の議論ではジャーナリスト（Z 氏）の一例を元に議論を展開していきましたが、広範なメディア単位でも表出しつつある課題でもあり、ミクロな観点でも一般のコミュニティ内でもソーシャル・ハラスメントとしてオンラインが利活用されるリスクは存在するため今後も議論を要する課題であると考えます。本稿では、分析対象としたSNS上のソースデータに関して被害者・加害者の両者のプライバシーに配慮をした考察がされている点に関しても（編集部了承済み）、今後の本議論の展開の方法論に関しての示唆も提示されており、より議論の深化に寄与されればと考えます。

 （2）追論すべきポイント

 本稿に関連するトピックとして、2021年11月31日にプレスリリースされましたが、Twitter上におけるなりすましに関するポリシー・利用規約が改訂されたことが今後、本議論でもどのように取り扱われるか、有効に働きうるかは課題です。

 “Twitter Safetyは「女性、活動家、反体制派、マイノリティコミュニティの一員に対し、過度に影響を与える可能性がある」とし、ポリシーに違反する画像や動画がシェアされた場合、Twitterはそのメディアを削除するとともにしかるべき強制的対応、たとえば当該ツイートを検索結果に表示されにくくしたり、ツイートの削除対応をしたうえで投稿者に通知し、またTwitterはポリシーに違反しているユーザーアカウントを永久に凍結する権限もあるとしています。” 意訳・引用元：https://twitter.com/TwitterSafety, 参照：2021/12/02)

 といった具体的な対策に関するケースも言及され、より一層本稿における議論は加速化すると考えております。

 主に、本稿でも示唆されている通り、ある“特定の個人”に不特定多数で、攻撃的な行為を行う、「成り済まし」を行った上で誹謗中傷を「暗に行う」事例などといった、陰湿なソーシャル・ハラスメント行為、所謂「ソーシャルポルノ」の事例も観測されております。

 また、搾取対象に対して無許可にメディア化する行為、身体的特徴を揶揄する行為、反社会的行為、つまりは個人の利益、日常行為を著しく害する行為などもSNS上で見受けられており、上述の引用のように注意喚起なども情報共有されつつある点ではプラットフォーム単位での環境改善は始まっているものの、まだこれから課題解決に働きかけが必要となる要素が多くあります。

 （3）本議論に関する今後の課題点、調査展望に関して

 本稿における課題に関しては、オンライン・ハラスメントの手法がテキストなど文面・ショートメッセージで発露するものから、トラッキングが困難になりやすい画像、イラスト、映像でのソーシャルポルノの暴露なども目立ってきている観点もあります。そのため、本論のようなケースに関して、さらに注視、また注意喚起などが教育機関での情報共有もなされるべきだと考えております。特に画像・音声メディア・映像など拡散性の高いメディアとなるとさらなるソーシャルポルノのリスクが発生し、被害者・加害者周辺における事実確認などが困難にもなりやすく隠蔽工作なども進みやすいところで、泣き寝入りとなるケースに関してもSNS上で被害者の声として観測されております。

 そのため、まずは問題意識の共有の契機は必要であると考えます。（上記のケースに関してはレビュワーによる匿名調査の元での見解。）そういった面で、学術的価値がある内容であると考えます。今後、より対策に講じるべき課題であり、これら問題解決をする際のSNS上での議論の過熱化、また加害者・被害者に対する合理的配慮、紛争解決の議論などを始めていくべきでもあり、インターネット上の違法・有害情報に対する対応（プロバイダ責任制限法）の改正なども本年度行われた観点も踏まえ、より学術的な議論も展開できればと考えるところです。また、本議論を展開するにおいてSNS上における「リスクマネジメント」など教育関係者での情報共有など、唐突な有害情報の暴露のリスクまで考慮にいれた議論、対応などもより必要なるかと考えます。そういった面で、本論における議論・論文は情報共有をする面でも有益に働く内容かとも存じます。

 （4）まとめ

 本稿でも触れている通り、ハラスメント防止策に関する提案に関して提示しているところ、インシデントの事例収集も必要になっていると考えます。

 特に、裁判など起こす力、知識を持たない若年層、または弱者など搾取対象とされやすい対象に対するインシデントは今後も発生しうると考えるため、これらの問題収集、対策の議論など情報共有、コミュニティ形成を推し量っていく必要もあるかと存じます。特に海外サーバーにあるプラットフォームにおけるインシデント・紛争解決は論文中でも触れてあるように解決までに時間を有することが多く、実効的な防止策に関する議論に関して継続的な試み、論文などにおけるジャーナリズムとしての意見共有の機会も必要かと考えるところ、本稿が一つの議論の深化に接続すればと願っております。

**参考文献**

 Twitter, Inc.: なりすましに関するポリシー. n.d. available at: https://help.twitter.com/ja/rules-and-policies/twitter-impersonation-policy. Accessed 2021/12/02.

 総務省: インターネット上の違法・有害情報に対する対応（プロバイダ責任制限法）. n.d. available at: https://www.soumu.go.jp/main_sosiki/joho_tsusin/d_syohi/ihoyugai.html

（該当する場合は要回答）統計分析および解釈は適切ですか。

対象外（統計を使っていない）

本研究は明確かつ正確に提示されたものであり、最新の文献を引用していますか。

はい

方法と分析について第三者による再現が可能となるよう十分な詳細が提示されていますか。

はい

結果の基礎となるソースデータはすべて入手可能で再現性を十全に保証していますか。

ソースデータは不要

結論は結果により妥当な裏付けを得ていますか。

はい

研究設計は適切で学術的価値がありますか。

はい

Reviewer Expertise:

SNS, 社会情報, メディア社会

I confirm that I have read this submission and believe that I have an appropriate level of expertise to confirm that it is of an acceptable scientific standard.

# Response

**Published:** 2022-01-13

**Authors:** Aki Tonami

**Affiliations:** University of Tsukuba, Japan

川畑先生

 査読コメントをいただき、誠にありがとうございます。

 いただいたコメントは、どれも大変重要、かつ今後の研究の発展に向けて参考になる指摘であると存じます。(2)(3)の追論のポイントに関しましては、今後の課題として、クロスプラットフォームで行われるハラスメントや、なりすまし、テキスト以外を使用したハラスメント行為の検討が必要として、追記いたしました。
